# Prediction and Control of Brucellosis Transmission of Dairy Cattle in Zhejiang Province, China

**DOI:** 10.1371/journal.pone.0108592

**Published:** 2014-11-11

**Authors:** Juan Zhang, Gui-Quan Sun, Xiang-Dong Sun, Qiang Hou, Mingtao Li, Baoxu Huang, Haiyan Wang, Zhen Jin

**Affiliations:** 1 School of Mechatronic Engineering, North University of China, Taiyuan, Shan'xi, People's Republic of China; 2 Complex Systems Research Center, Shanxi University, Taiyuan, Shan'xi, People's Republic of China; 3 School of Mathematical Science, Fudan University, Shanghai, People's Republic of China; 4 The Laboratory of Animal Epidemiological Surveillance, China Animal Health & Epidemiology Center, Qingdao, Shandong, People's Republic of China; 5 Department of Mathematics, North University of China, Taiyuan, Shan'xi, People's Republic of China; 6 School of Mathematical & Natural Sciences, Arizona State University, Phoenix, AZ, United States of America; Imperial College London, United Kingdom

## Abstract

Brucellosis is a bacterial disease caused by brucella; mainly spread by direct contact transmission through the brucella carriers, or indirect contact transmission by the environment containing large quantities of bacteria discharged by the infected individuals. At the beginning of 21st century, the epidemic among dairy cows in Zhejiang province, began to come back and has become a localized prevalent epidemic. Combining the pathology of brucellosis, the reported positive data characteristics, and the feeding method in Zhejiang province, this paper establishes an 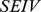 dynamic model to excavate the internal transmission dynamics, fit the real disease situation, predict brucellosis tendency and assess control measures in dairy cows. By careful analysis, we give some quantitative results as follows. (1) The external input of dairy cows from northern areas may lead to high fluctuation of the number of the infectious cows in Zhejiang province that can reach several hundreds. In this case, the disease cannot be controlled and the infection situation cannot easily be predicted. Thus, this paper encourages cows farms to insist on self-supplying production of the dairy cows. (2) The effect of transmission rate of brucella in environment to dairy cattle on brucellosis spreading is greater than transmission rate of the infectious dairy cattle to susceptible cattle. The prevalence of the epidemic is mainly aroused by environment transmission. (3) Under certain circumstances, the epidemic will become a periodic phenomenon. (4) For Zhejiang province, besides measures that have already been adopted, sterilization times of the infected regions is suggested as twice a week, and should be combined with management of the birth rate of dairy cows to control brucellosis spread.

## Introduction

Brucellosis, also called Bang's disease, Crimean fever, Gibraltar fever, Malta fever, Maltese fever, Mediterranean fever, Rock fever, or Undulant fever, is a highly contagious zoonosis caused by brucella. In China, it is usually called “Lazybones disease”. It is listed in Class B animal epidemics by the World Organisation for Animal Health (OIE) and Class II as one of the notifiable diseases by the Law on Prevention and Control of Infectious Diseases of the People's Republic of China [1]. In 1985, according to the characteristics of brucella and host specificity, the committee of WHO divided Brucella into 6 species, that is *B. melitensis* (goats and sheep), *B. suis* (pigs), *B. abortus* (cows and bison), *B. ovis* (sheep), *and B. canis* (dogs) [2]. For cows, *B. melitensis*, *B. suis*, *B. abortus* are found, and *B. abortus* is the dominant species [3]. *B. abortus*, *B. melitensis*, *B. suis*, and *B. canis* are pathogens to invade human. Brucella is highly contagious, and can be spread by direct contact transmission through the brucella carriers or indirect contact transmission when animals ingest contaminated forages or the excrement containing large quantities of bacteria, generally discharged by infected animals. The brucella can survive 20-120 days in soil, 70–150 days in water, and 2 months in food such as milk and meat [4]. However, the brucella can be killed easily by direct sunlight within a few hours or by high temperatures within a few minutes. Once infected by brucellosis, animals should go through 14–180 days incubation [4] until they show symptoms.

The finding of brucella carriers, for dairy cows, mainly depends on regular detection. Diagnosis methods during the detection process are based on bacteriology or serology, which generally includes screening tests and supplemental tests. Screening tests are used to locate infected population, but there is a high percentage of false positive results. So, supplemental tests, which include complement fixation, rivanol precipitation and milk samples test, are used to clarify the results of screening tests [5]. However, there also exist some false negative herds not to be detected by tests. It follows that these diagnosis are not completely effective and only about 84%–98% of infected dairy herds can be detectable [6]. However, whether the dairy herd in incubation can be detectable is an outstanding issue.

Zhejiang province is located on the southern part of China, in which there are hills, mountains, basins, islands and the Qiantang river. It belongs to subtropical monsoon climate area with four entirely different seasons, plentiful sunshine and rainfall. Thus, the farming there has experienced extensive development. In 1950s, that Hangzhou, Ningbo, Huzhou city in Zhejiang province imported infected dairy cows from north provinces made the Zhejiang province develop a brucellosis prevalence among dairy cows. From 1983 to 1995, Zhejiang province carried out brucellosis general census and culled all the detected positive reactors of livestock [7]. Consequently, in 1995, the brucellosis epidemic in Zhejiang province reached control standards of government regulations. However, at the beginning of the 21st century, the epidemic situation between livestock, especially dairy cows, began to come back, even increased year by year and has lead to the local prevalence of brucellosis [8]. In 2001, there was only 14 infected dairy cows. Quickly, it increased to 248 in 2005 and 527 in 2009, respectively. In 2010, the accumulative number of the infected dairy cows arrived 1808. Simultaneously, the diagnosis methods taken in Zhejiang province were serological examination: tube agglutination test (SAT) and complement fixation test (CFT) [8]. Though culling the reactors and regular detection are taken powerfully, the positive data of dairy cows in Zhejiang are rising year by year, which has negatively influenced the local economy, even leads to the local prevalence of human brucellosis. By the full-survey and analysis for brucellosis in Zhejiang province, some crucial factors are found to interpret the spread of brucellosis. Firstly, during the past ten years, the livestock breeding, dairy, and the leather processing industry had experienced great development. A mass of dairy cows, beefs, row furs and other animal by-products were imported from the northern pastoral areas annually. When the brucellosis in the northern pastoral area began to return, and the imported cows from northern areas cannot get effective quarantine inspection and were directly mixed with local cows in Zhejiang [8], brucellosis was brought into Zhejiang province. Secondly, culling measure cannot be carried out completely and effectively, so the sources of infection are not removed thoroughly [8]. Lastly, the sensitivities of the surveillance and confirmation tests were not 100%, collected from the literature as follows: 84% for the SAT [9], [10] and 89% for the CFT [9], [11]–[13]. Moreover, there exists improper handling in practical culling and test processes. So, some infected dairy cows cannot be detectable and will become hidden dangers to cause the spread of brucellosis. It is thus clear that the recent reemergence of brucellosis in dairy cows poses a serious threat on the economy and public health in Zhejiang province and we need resort to dynamical modeling to explore the inherent factors of brucellosis transmission and assess prevention and control measures.

Mathematical models which are applied to investigate epidemic spreading have various forms, such as dynamical systems, statistical models, game theoretic models and so on, where the dynamical system is a very important method, whose main idea is to build evolution rule that describes how future states follow from the current states. Therefore, the dynamical system shows inherent link and internal change pattern of sub-populations, and can be applied to forecast future states of disease in considered populations. So far, dynamical models have been adopted to explore the transmission dynamics of brucellosis. In 1994, Gonzalez-Guzman and Naulin [14] built a model about bovine brucellosis, used singular perturbations method to analyse the dynamical behavior, and obtained a threshold parameter for the outbreak of the disease. In 2009, Xie and Horan [15] built a simple dynamical model, including the susceptible, the infected, and the resistant subclass, to discuss brucellosis in elk and cow populations. They mainly investigated private responses and ecological impacts of policies, and found feedbacks between jointly determined disease dynamics and decentralized economic behavior matter, whose novel point is to combine disease with the economic factor. Because hosts can also be infected by a contaminated environment, Ainseba et al. [16] considered two transmission modes about the ovine brucellosis: direct mode caused by contact with infected individuals, and indirect mode related to the presence of virulent organisms including brucella in the environment, which we think is a dominant and important factor for brucellosis transmission. They obtained the reproduction number, and investigated the effect of a slaughtering policy. Xie and Horan [15] and Ainseba et al. [16] only investigated the transmission dynamics of brucellosis between animals. In 2005, besides transmission within sheep and cow populations, Zinsstag et al. [17] considered the transmission to humans to fit demographic and seroprevalence data from livestock and annually reported new human brucellosis cases in Mongolia from 1991 to 1999. The livestocks are classified into three subclasses: the susceptible, the seropositive, and the immunized. They mainly showed that average effective reproductive ratios were 1.2 for sheep and 1.7 for cows. However, there is very few research to study the brucellosis in China which is more serious. Hou et al. [18] investigated the transmission dynamics of sheep brucellosis in Inner Mongolia Autonomous Region of China and discussed the effects of vaccination, disinfection and eliminating strategies between young and adult sheep.

In 2011, while in collaboration with China Animal Health & Epidemiology Center in Qingdao, we went to Zhejiang province to carry out field research. We mainly visited dairy cow raising areas and communicated with local farmers and government, by which the detailed information about dairy cow breeding was obtained. The breeding mode for dairy cows is mainly large-scale raising zone construction. Zhejiang province imported some fine varieties of dairy cows from northern areas every year. As a result, the input of dairy cows has brought brucellosis into Zhejiang province. According to the breeding mode, control measures in Zhejiang province, the model established in this paper has five characteristics. Firstly, since the breeding mode for dairy cows in Zhejiang province is mainly large-scale raising zone construction, so standard incidence rate is adopted. Secondly, for dairy cattle in Zhejiang province, taking safety of milk products into consideration, vaccination for cattle is not carried out. So, it is not included in our model. Thirdly, since there are 14–180 days incubation period for cattle brucellosis, the exposed subclass is considered. Individuals in the period of incubation are hardly detectable, but can infect the healthy dairy cows by direct contact or by discharging brucella to environment, thus the consideration for incubation period is necessary. Moreover, the import of cattle from north areas is considered, since it is the main reason of prevalence of brucellosis in Zhejiang. Finally, there exist two transmission modes for brucellosis: direct transmission between individuals and the transmission of infected environment. Since dairy cows are infected mainly through ingesting contaminated forages or the excrement discharged by infected animals, the infection through environment is vital and indispensable, even more important than the direct contact transmission between the individuals. For previous models, [15]–[18] adopted bilinear incidence rate and considered immunized groups. Environment transmission did not be considered in [14], [15], [17]. The import of individual from other areas was not considered by [14], [15], [17], [18]. Based on these literatures, this paper builds an SEIV model with external input of dairy cows to fit the real situation, reveal the transmission mechanism, predict the spread situation and assess control measures of dairy cattle brucellosis in Zhejiang province in China. After 2000, there is more relatively regular and standard management in Zhejiang province: large-scale raising zone construction, regular inspection and positive-cull. The detailed data and information can be obtained. Studying the situation in Zhejiang province can provide suggestion and revelations for whole cattle management and epidemic control in China.

In addition, there are three points that need to supplement. Firstly, for brucellosis, prevention and control measures, that is detection twice a year and 100% culling of the detected positive cows that have already been adopted in the Zhejiang province are, are all considered in our model. Moreover, the discharge rates of amount of brucella between the exposed and infectious is assumed to be the same. In fact, the amount of brucella discharged by the exposed and infectious should be different. In order to distinguish the difference between the exposed and infectious, a supplementary parameter should be introduced. As we know, the discharge rate is hard to quantify and the supplementary parameter is also uncertain. So, it makes little sense to distinguish the difference of amount of brucella discharged by the exposed and infectious that can increase the number of uncertain parameter and has little effect on analysis result. Finally, we obtain the positive data of dairy cows in Zhejiang province which are reliable and sufficient, and can use them to confirm the validity of the dynamical model.

## Methods

### Dynamical model

**Figure 1:**
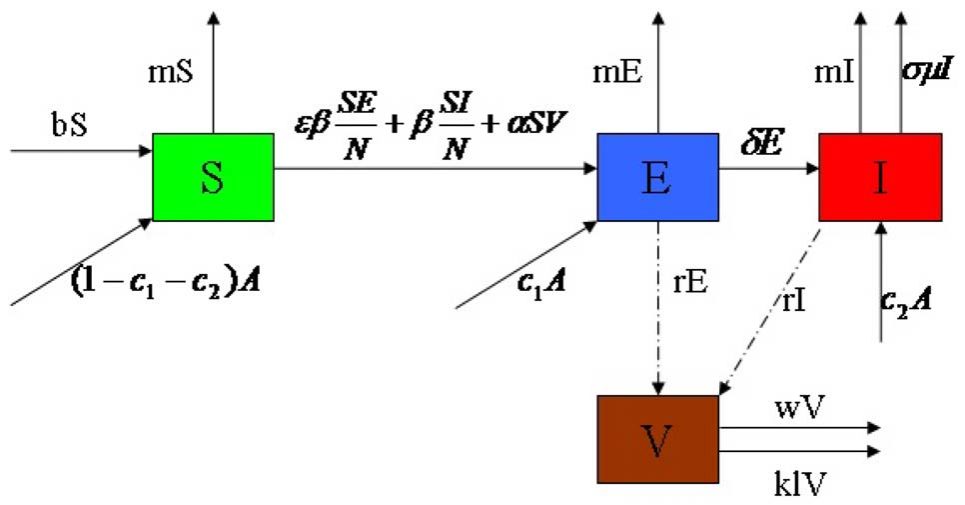
Flow diagram on the dynamical transmission of brucellosis among dairy cattle. Solid arrows represent transfer orientation of population (the department from one subclass and incoming to another subclass). For a subclass, the incoming solid arrows (point to the subclass) represent the recruitment of cattle to this subclass, and the outgoing solid arrows (point to other subclass) represent the runoff of cattle or brucella from this subclass. Dash arrows only represent incoming of brucella to environment discharged by 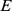 and 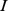.

The populations we consider in the model are the dairy cows and the brucella contained in excreta discharged by infected dairy cows into environment. Let 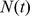 be the total number of dairy cows in Zhejiang province under consideration at any time t, which are classified into three subclasses: the susceptible, exposed and infectious denoted by 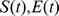 and 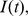 respectively. The quantity of brucella in environment is denoted by 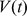. With regard to 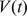, it is very difficult to determine the quantity in environment and the quantity that is enough to infect an individual. Hence, in this paper, we define that the average number of bacteria that are needed to infect a host with brucellosis is called *an infectious unit* [18]. Thus the unit of 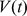 is *the infectious unit*, shortly “*IU*”. The mathematical model to be discussed is to study the rate of change of the all populations, especially the infectious dairy cows. Our assumptions on the transmission process of brucellosis among dairy cows are demonstrated in the following flowchart (Fig. 1). According to Fig. 1, the deterministic dynamic model is given as follows, where parameters are described in Table 1. The detailed description of model is given in Appendix S1.
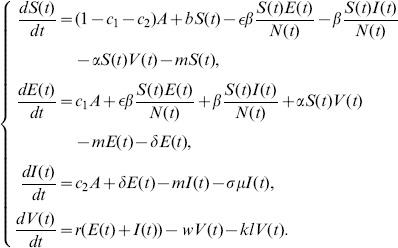
(1)


**Table 1:** Description of parameters in the model (1) and (2).

Parameters	Value	Unit	Comments	resource
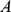		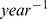	Annual introduction number of dairy cow.	estimating
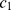	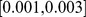	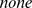	The proportion of the exposed on A.	assuming
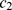	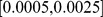	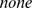	The proportion of the infective ion A.	assuming
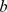	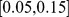	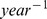	Annual birth rate of S(t).	assuming
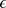	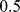	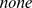	Auxiliary parameter.	assuming
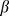		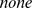	I(t)-to-S(t) transmission rate.	estimating
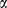		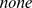	V(t)-to-S(t) transmission rate.	estimating
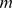	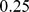	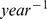	Dairy cow natural elimination rate.	[6]
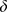	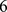	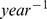	Clinical outcome rate of exposed cows.	[4]
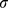	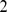	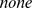	The frequency of annual quarantine.	[a]
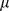	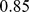	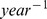	The culling rate of I(t) after quarantining.	[9]–[13]
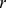	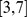	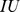	The discharging quantity of brucella by 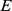 and 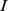 .	assuming
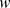	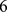	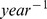	The natural death rate of V(t).	[4]
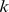	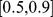	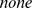	The effective disinfection rate each time.	assuming
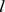	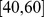	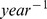	The frequency of disinfection annually.	assuming
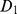	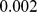	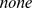	The intensity of white noise.	fitting
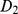	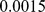	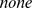	The intensity of white noise.	fitting
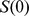	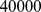	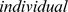	The initial number of the susceptible cows.	[19]
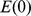	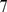	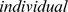	The initial number of the exposed cows.	assuming
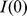	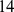	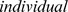	The initial number of the infective cows.	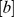
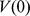	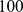	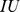	The initial quantity of brucella.	assuming

[a] Field research and personal communication. [b] The Laboratory of Animal Epidemiological Surveillance, China Animal Health & Epidemiology Center.

Due to the complexity of system, we just give limited results about dynamical behaviors in Table 2, whose related theorems and proofs can be seen in Appendix S1, and related Figures are given as Figs S1, S2, S3 and S4, where 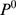 is the disease-free equilibrium, 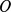 is zero equilibrium, 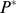 is endemic equilibrium, and 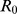 is the basic reproduction number [20], [21].

**Table 2:** Equilibria and stability.

Cases	Conditions	Equilibria	Stability	Possible steady state
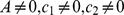		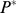	Not proved	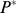 or limit cycle
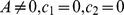	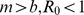	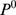	G.A.S.	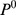
	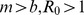	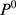 , 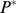	uniformly persistent	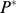 or limit cycle
	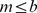	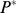	Not proved	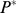 or limit cycle
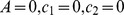	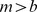	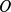	G.A.S.	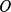
	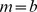	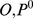	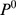 is stable	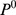 .
	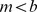	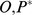	Not proved	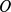 or 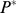 or limit cycle

### The Interpretation of Parameter Values

Now, we interpret the parameter values as follows. Due to limited data, we have to make assumptions about 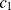, 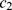, 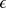, 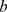, 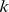, 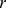, 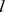, 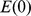, and 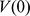, where 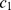, 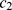, 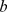, 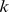, 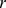 and 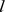 are taken as random numbers from certain reasonable intervals. Recently, the number of cattle in Zhejiang province is between 40000 and 80000 and the number of infected cattle is from 100 to 600, so the infected rate is about 0.00125 to 0.015. So, we assume that 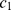 is between 0.001 and 0.003 and the value of 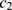 is less a little than 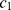. For b and r, we give the corresponding reasonable intervals by fitting real data. k is disinfection effective rate and should be between 0 and 1. 100% is impossible and the disinfection measure is taken well in Zhejiang province, so we assume the interval is from 0.5 to 0.9. l is the disinfection frequency. According to personal communication, breeding field is disinfected about once a week. There are 52 weeks in one year. So, l is taken random number between 40 and 60. 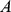, 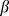 and 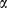 are obtained by parameter estimation. 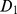 and 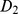 are obtained during fitting data by stochastic model to make the perturbation amplitude of solution consistency with the actual data. 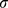 is assumed according to the personal communication of authors during field research. In Zhejiang province, government carries out surveillance tests twice a year, and the unit of time taken in the model (1) is one year, so 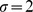. Because the sensitivities of tube agglutination test (SAT) and complement fixation test (CFT) adopted as the diagnosis methods are 84% and 89%, we assume the average sensitivity is 85%. Moreover, Zhejiang province culls all the discovered positive reactors, so the culling rate for the infected herd is 85%, that is 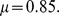 The detailed introduction of basic knowledge about dairy cows can be seen in [6]. A heifer(dairy cow) reaches sexual maturity within 18 months of age and it will have no reproduction value after the third pregnancy [6]. So, the dairy cow is removed from the herd at four years of age on average, that is 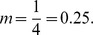 According to [4], for dairy herds, the brucella has a 14–180 day incubation period. Here we take 2 months, so 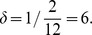 Besides, the brucella can survive 20–120 days in soil, 70–150 days in water, 2 months in food such as milk and meat. So, we take the mean value 2 months, that is 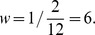 The initial number of dairy cows 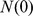 in Zhejiang province is taken from [19], which can be viewed as the initial number of the susceptible cows 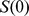 for the numbers of 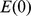 and 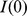 are very few relatively. The data of 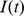 from 2001 to 2010 is provided by China Animal Health and Epidemiology Center in Qingdao city.

### The least-square estimated method

We apply the least-square method to carry out parameter estimation, which is implemented by the command *fminsearch*, a part of the optimization toolbox in MATLAB. Let 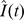 be the real data and 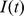 is the theoretical result of model. The estimation method is to find parameter values such that the value of 
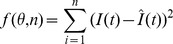
 is the least, where 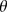 is the parameter vector and 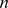 is the number of real data.

## Results

### Numerical Fitting and Prediction

**Figure 2:**
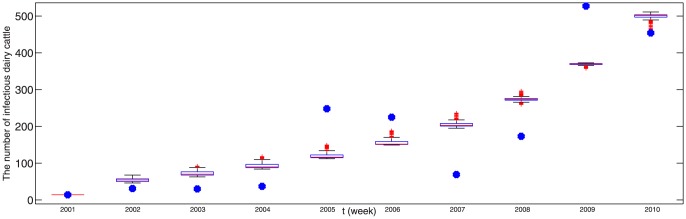
100 times of fitting results of positive data in Zhejiang province by deterministic model, where blue dotes are real data and the boxplot is the result of model (1). The parameter values taken in simulation are given in Table 1 and the estimated parameter values are given in Table 3.

The real new data about infected dairy cattle and cattle population are listed in Tables S1 and S2 in Appendix S1, respectively. By running 100 times, the estimated parameter values are listed in Table 3 and corresponding fitting results by deterministic model are given in Fig. 2, where the red dots is the real number of annual new infectious cattle in Zhejiang province and the boxplots is drawn by applying 100 times of simulation results by deterministic model. It is obvious that real data have certain fluctuation and the theoretic fitting result of deterministic model is general trend of the epidemic.

**Table 3:** Values of estimated parameters.

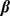		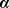		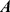	
mean value	CI	mean value	CI	mean value	CI
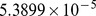	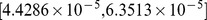	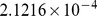	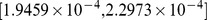	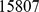	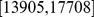

**Figure 3:**
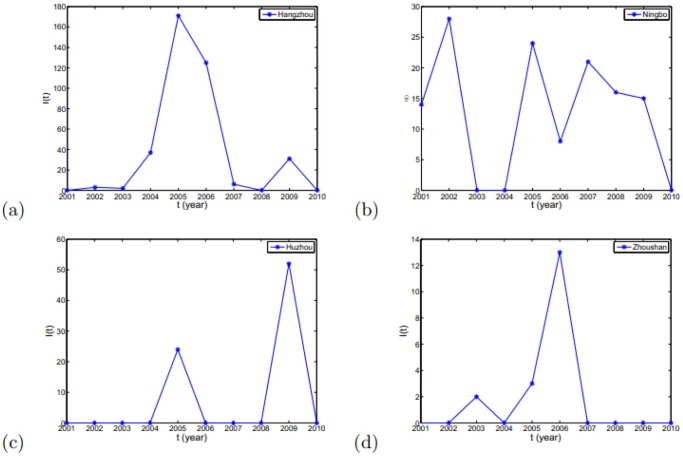
The real positive data of cows infected with brucellosis in Hangzhou city, Ningbo city, Huzhou city and Zhoushan city in Zhenjiang province. (a) Hangzhou city. (b) Ningbo city. (c) Huzhou city. (d) Zhoushan city. These data are listed in Table S1 in Appendix S1.

Recently, the government in Zhejiang province has intensely taken powerful quarantine inspection and slaughter of the positive reactors to control brucellosis. However, there still remains the positive dairy herds in the process of the quarantine inspection. Based on the literatures and full-survey in Zhejiang province, it is known that the most crucial factor which leads to the prevalence of brucellosis is the import of infected dairy cows from the the northern pastoral areas, which are the high-prevalence areas. However, the import does not get effective detection, which leads to the input of the infected dairy cows [8]. Moreover, the positive data in several cities in Zhejiang province had randomness, which can be seen in Fig. 3. Particularly, in Fig. 3(c), Huzhou city always was exempt from brucellosis before 2004. However, in 2005, suddenly 25 cows tested positive, then all of them were culled and disposed of. During the following three years, Huzhou city was free of brucellosis infection in dairy cows and had no infection source. However, in 2009, the positive cases reappeared. The interpretation of this ruleless phenomenon can be the randomness of input of the infectious dairy cows from the outsides.

**Figure 4:**
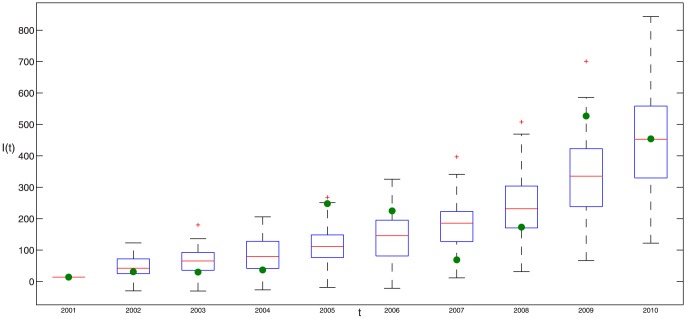
100 times of fitting result of positive data in Zhejiang province by stochastic model (2), where green dotes are real data and the boxplot is the result of model (2). Here 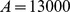,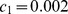, 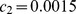, 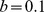, 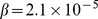, 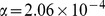, 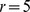, 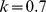 and 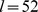. Other parameter values and initial values of variables taken in simulation are given in Table 1.

**Figure 5:**
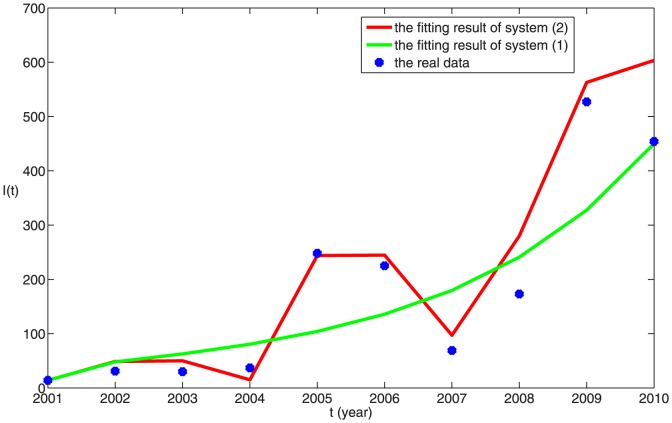
The comparison between the reported positive dairy cows in Zhejiang province in China from 2001 to 2010 and the simulation results of 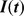 in models (1) and (2), which is one of the best fitting results. The blue dots represent the reported data. The green curve is the solution of system (1) and the red curve is an example of a simulation of system(2). Here 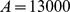,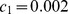, 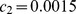, 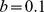, 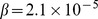, 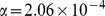, 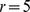, 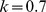 and 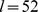.

**Figure 6:**
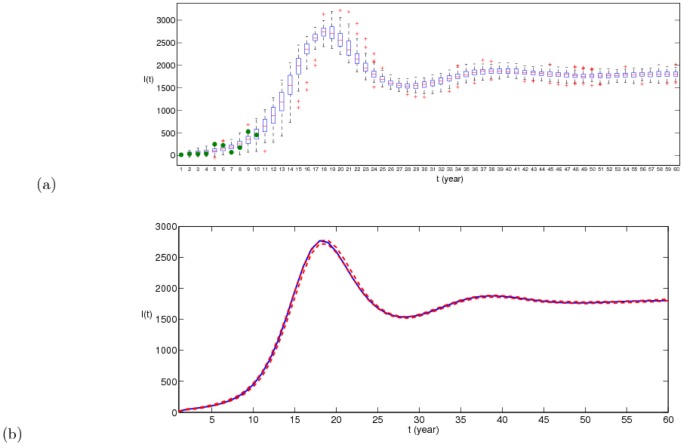
100 times of prediction results of 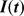 in models (1) and (2) during 50 years. (a) in model (2); (b) in model (1), where blue curve is the mean values and the two red dotted curves represent 95% confidence interval.

Thus, in order to more precisely fit the data of positive cases in Zhejiang province, we need to resort to the stochastic dynamic model, which can be obtained by adding stochastic perturbations to some parameters in model (1). Because the randomness is caused by the input of the brucellosis carriers. So, we add stochastic perturbations to 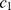 and 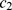. So, the model (1) is rewritten as the following form to fit the real data:
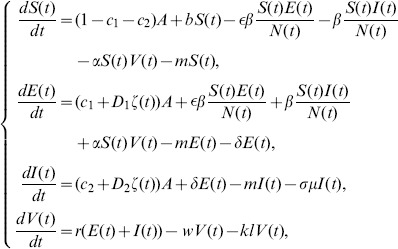
(2)where, 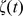 is a time series of random deviates derived from the normal distribution with mean zero and unit variance; 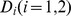 represents the intensity of 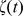. In this case, 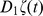 and 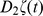 also can be reviewed as stochastic perturbations to 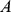 if adding the first three equations together. Next, we use this stochastic dynamic model to fit the positive data of the infective dairy cows in Zhejiang provinc. The parameter values and the initial values adopted in fitting are listed in Table 1 and some parameter values (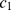, 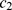, 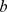, 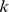, 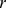 and 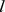) are fixed to be cosntant. During the simulation, what is difference with deterministic model is to add perturbations(white noise) 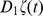, 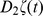 to the parameters 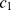 and 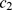, respectively, where 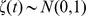. Due to the stochastic perturbations, the fitting result is different when we run the program about the stochastic dynamic model every time. 100 times of fitting result are given in Fig. 4, where one of the best fitting result is given in Fig. 5. The good fitting result demonstrates our mathematical model has certain rationality, so we can use it to predict the disease situation and assess the prevention and control measures. The prediction trend of brucellosis in Zhejiang province by deterministic model and stochastic model are shown in Fig. 6 (a) and (b), respectively. From Fig. 6(a), with randomness of import of infected dairy cows, the prediction situations can have large differences that can reach up to several hundreds. When the system attains steady state, the number of positive cattle will be between 1500 and 2000. From Fig. 6(b), we can see that brucellosis cases of the dairy cows will steadily increase in the next 17 years and arrive a peak(about 2700), then decrease to 1500 and lastly tend to a steady state about 1700, which is mean value in Fig. 6(a). By applying deterministic model, we can obtain the general and mean trend of epidemic. By applying stochastic model, although ruleless perturbation are added, the cases range of dairy cattle can be obtained. From Fig. 6, it can be seen that if not to take more efficient measures, the disease in dairy cows cannot be controlled. With the randomness of import of brucellosis carriers, the situation in Zhejiang province cannot be precisely predicted. So, firstly we encourage Zhejiang province to take self-supplying of dairy cows, rather than the importation of dairy cows from north areas, which is the first conclusion in this paper.

### Control Measures Assessment


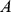
 is the number of import cattle and its effect on disease is effect of the import number. 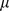 is the culling rate of positive cattle and its effect on disease is effect of culling measure. 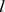 is the frequency of disinfection and its effect on disease is effect of disinfection measure. 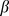 is the cattle-to-cattle transmission rate and its effect on disease is effect of direct contact rate. 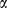 is the brucella-to-cattle transmission rate and its effect on disease is effect of indirect contact rate (environment contact rate). 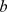 is the birth rate and its effect on disease is effect of the birth number. c1 and c2 are the proportions of exposed and infectious cattle on the whole import cattle, respectively. c1 and c2 are related with the detection strength for the import cattle. When detection rate is larger, c1, c2 may be smaller.

**Figure 7:**
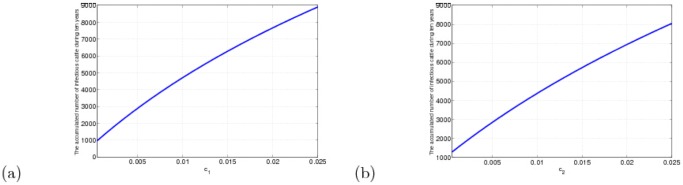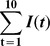 in term of 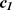 and 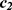. (a) 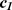. (b) 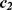. Here 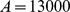, 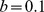, 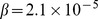, 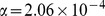, 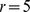, 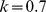 and 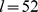.

**Figure 8:**
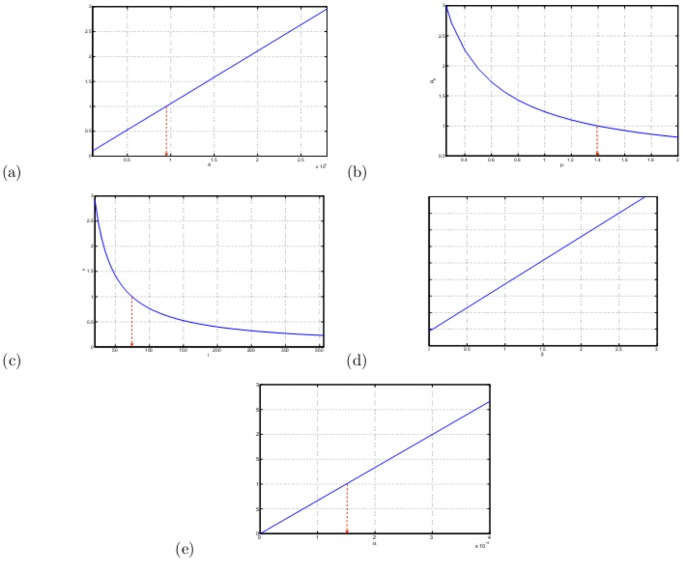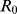 in terms of 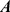, 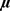, 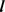, 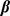 and 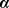. (a) 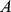. (b) 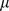. (c) 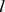. (d) 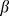. (e) 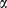.

The influences of 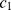 and 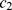 on disease spread are relatively large, whose effects on the accumulated number of infectious cattle during ten years (2011-2020) are given in Fig. 7. Compared Fig. 7(a) with Fig. 7(b), it is easily to know that the effect of 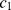 is larger. When 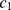 varies from 0.0005 to 0.02, the accumulated number of the infectious cattle will rise to 9000 from less than 1000. If the same changes are taken for 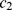, the accumulated number of the infectious cattle will rise to 8000 from more than 1000. Therefore, firstly and the most important, the detection strength should be enhanced and the import of the brucella carriers must be completely eradicated, or the disease cannot be removed and predicted precisely. Let 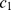 and 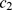 equal to zero, we can calculate the basic reproduction number 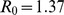, whose calculation can be seen in Appendix S1. At the same time, we can carry out sensitivity analysis of some crucial parameters, see Fig. 8. From the five figures, we can see that 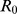 is linear in term of 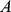, 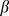 and 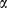, concave function in term of 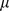 and 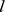. Observing the shape of curves and the ordinate axes, it can be easily known that when 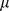 and 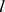 is smaller, their influences are bigger than 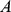, 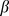 and 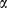. When they increase, the influences become smaller and smaller, especially, when 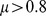 and 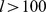. In addition, according to the current situation, 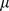 cannot make 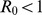, even 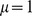, that is all the infected dairy cows are culled, which is because of the existence of and the exposed subclass and environment transmission. The culling measure, that is 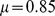, adopted by Zhejiang province is enough. With regards to Fig. 8(c), sterilization once a week, that is 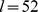, can be changed into twice a week, that is 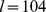, which is enough. However, the two measures cannot remove the brucellosis in dairy cows. So, the influence of 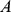 should be considered. If 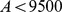, 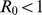, which can make the disease disappear. Otherwise, the disease will be persistent. Next, it is necessary to compare the influence of 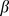 and 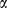. The decreasing of 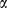 and 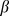 can both reduce 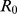. When 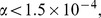 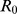 will be less than 1. Sensitivity of 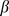 is less than 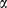 and Even 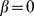 cannot make 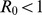, so the prevalence of brucellosis is mainly aroused by the environment transmission, and it should arouse more attention of government and farmers. Thus, that the effect of infection of brucella in environment on brucellosis spread is greater the infection during individuals is the second result obtained in this paper.

**Figure 9:**
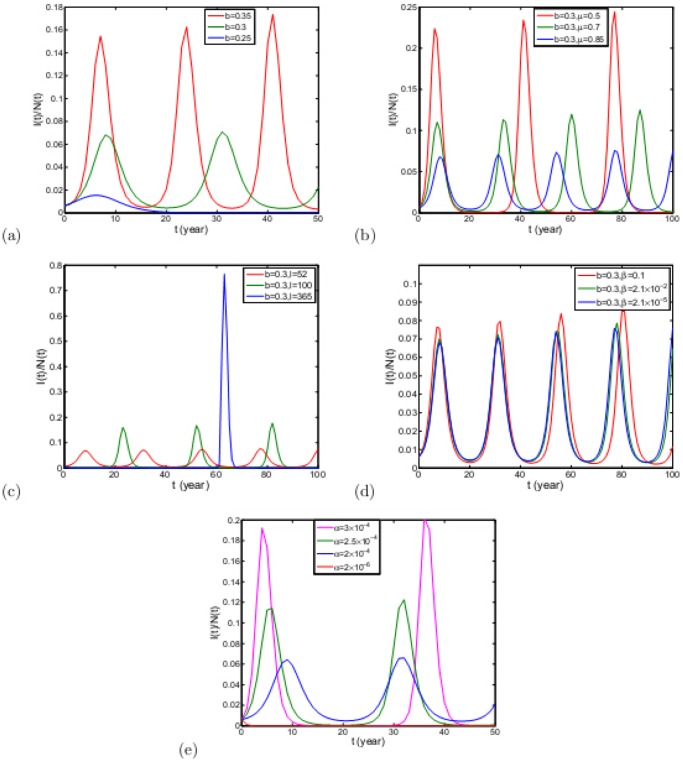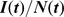 with 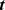 under different 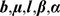. (a) 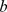. (b) 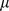. (c) 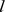. (d) 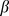. (e) 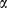. The initial values of variables can be seen in Table 1.

As we know, due to constraint of sensibility of detection methods, as long as there are dairy cows to import, the input of infected dairy cows is unavoidable. When 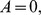 that is insisting on self-support, the disease situations under different conditions are shown in Fig. 9. Because the positive rates of dairy cows is more concerned, we give the solution curves of 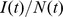 with 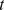, not 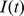. From Fig. 9, we can know that under some conditions, there must appear periodic cycles, which is interesting result of this paper. Firstly, let us look at the prediction of the positive rate under different 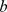. If Zhejiang province insists on self-support, 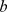 must be not less than 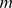, 0.25, or the total number of dairy cows will go to zero. When 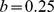, from the second result of Theorem 4, there is only one disease-free equilibrium. So, the brucellosis in Zhejiang province will disappear in 20 years, see Fig. 9(a). From the Fig. 9, when 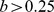, the disease cannot disappear. If 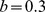, the disease is prevalent and will be periodic with period of 20 years. Meanwhile, its positive rate circulates between 0.005 and 0.065. With increasing 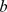, the positive rate will increase and the period will be shortened. From Fig. 9(b), we know that 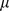 has large effect on disease control. When 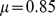, the disease is also prevalent with period of 20. When 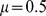, the period becomes 30 and the positive rate can reach up to 0.25. So, with the increase of 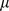, the positive rate will decrease, however the period will be shortened. On the contrary, to increase 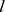 can shorten the period, which can reduce the disease situation temporarily. In 60 years, the positive rate is very low. However, once the disease outbreaks, the peak of wave becomes very high. From Fig. 9(d) and (e), it is easy to see that the influence of 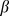 on positive rate is less than 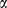. When 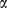 increases, the period will enlarge, and the positive rate will rise. When 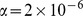, the disease can be controlled temporarily. In conclusion, in order to eliminate disease thoroughly under insisting on self-support, government of Zhejiang province should control the birth rate 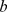 to be 0.25. If keeping the birth rate 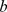 be more than 0.25, it is necessary to control disinfection frequency 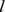. It must be important to notice the influence of 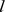. According to the common sense, the increasing of 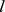 can lead to relief of the disease, which can give us more time to find more effective measures. However, the peak of wave will increase with the growth of 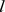, which should draw our attention as well. So, from Fig. 8(c), we suggest that twice a week is enough, which is another interesting and important result of this paper.

### Uncertainty Analysis

**Figure 10:**
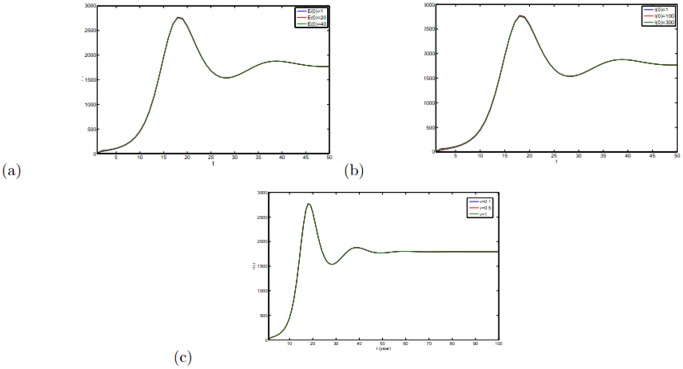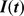 with 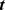 under different initial values and parameter 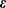. (a) under different 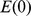. (b) under different 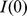. (c) under different 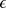. The initial values of variables can be seen in Table 1.

Due to the lack of information, some initial value and parameter value are assumed and given roughly. Now, the effect of initial values 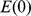,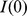 and parameter 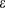 on the epidemic situation are given in Fig. 10. From Fig. 10, it can been easily to see that 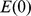 and 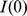 have little influence on the epidemic situation. 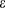 has no influence on epidemic spread, since the effect of direct transmission between individuals on epidemic spread is little.

**Figure 11:**
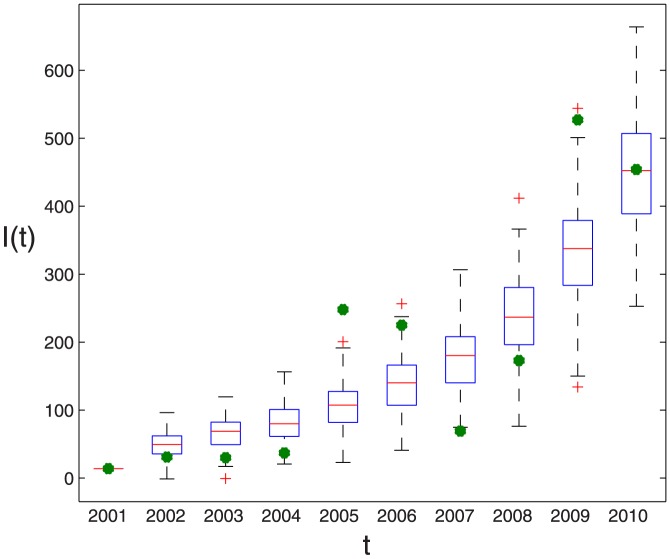
100 times of fitting result of positive data in Zhejiang province by stochastic model (3), where green dotes are real data and the boxplot is the result of model (3). The parameter values and initial values of variables taken in simulation are the same as Fig. 4.

In addition, if we only add stochastic perturbations to 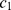 and 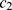 and the number of import is constant. So, the model (2) is rewritten as the following form, whose fitting result is given in Fig. 11 and has little difference with Fig. 4. Thus, the model (2) and the model (3) are all right and both cases can be adopted to study cattle brucellosis in Zhejiang province.
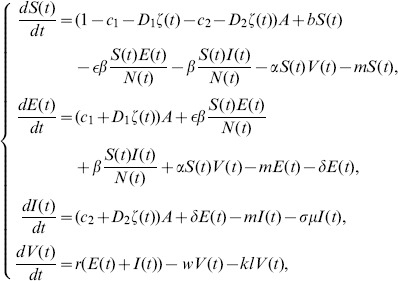
(3)


## Discussion

Brucellosis is a notifiable disease that can infect cows, swine, goats, sheep, dogs and humans. In the Zhejiang province, brucellosis between dairy cows has attracted significant attraction of the public and government for it is a major public-health and economically devastating zoonosis. Although recently, the Zhejiang province has taken strict regulations to control brucellosis: the detection twice a year and culling all the infectious cows, however it is still a threat to the health of milk products and the development of breeding businesses. So, according to the transmission mechanism of brucellosis in dairy herd and the breeding business characteristic of Zhejiang province, this paper builds an 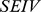 dynamical model to investigate the internal transmission dynamics, predict the infection situation, and assess prevention and control measures. Currently, efforts are suggested to direct at regular detection and eliminating reactors [22]–[24], prevention(vaccination) [23], [25] and sterilization [23]. Applying the dynamical model, this paper analyzes and forecasts the disease's behaviors with the quantitative point of view. After obtaining the corresponding precise parameter values, we can give the strength of measures to be needed to control the disease transmission.

For Zhejiang, the greatest danger comes from imported animals. With regulation, they should import dairy cows from brucellosis-free areas, and herds should be isolated for 30 days and retested to be seronegative before being blended with the local herd. However, in practice these are not well carried out. Moreover, the effective rate of test cannot reach up to 100%. It is concluded that import of cattle can make that the prediction situations can have large differences that can reach up to several hundred. So, firstly we encourage Zhejiang province to take self-supplying of dairy cows, rather than the importation of dairy cows from north areas. During intervention measures, elimination of seropositives is more discussed by [14], [16], [17], since it is more effective to reduce brucellosis prevalence in cattle. Gonzalez-Guzman and Naulin [14] gave the necessary rate of elimination of seropositives to control disease. [17] drew a conclusion that the test-and-slaughter intervention appeared more effective to reduce brucellosis prevalence in cattle than the vaccination scenarios. For Zhejiang province, the regular detection, eliminating reactors, and disinfection of environment in local herds have been implemented well: testing twice a year and 100% culling. Generally, herds must be tested at regular intervals until 2 or 3 successive tests are negative. To increase the frequency of tests annually will cost too much time and money, and it is strenuous. Though the frequency of detection for dairy herds has better results at once a month [6], twice a year that is taken by Zhejiang province is enough. Slaughtering policy has been carried very well and is not considered and discussed in this paper. Ainseba et al. [16] concluded that the contamination of the environment can play an important role in the persistence of disease. In this paper, by simulation analysis, sterilization is a measures we should use carefully to control brucellosis in Zhejiang province. When 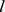 is larger, although brucellosis can be controlled temporarily, once the brucellosis appears, the disease must be a large outbreak. Since the complexity of the disinfection times, this measure should always be adjusted with regards to real circumstance. [17] gave simulated vaccination scenarios. However, vaccination of dairy herds can increase resistance to infection, however resistance may not be complete, and some vaccinated individual may become infected, depending on severity of exposure. Therefore, vaccination has many indeterminacies and needs more discussion [5], here it is not proposed. From Table 2, according to the current breeding model in Zhejiang province, we can know that under certain condition cattle brucellosis may be periodic and the cycle can be from 15 to 20 years long (Fig. 9), which was not found and discussed in previous literatures. According to the period, related government should not let their guard down and can propose corresponding prevention measure. In addition, by dynamical and sensitivity analysis, we suggest government to control brucellosis by managing the birth number of calf. All these control measures can reduce economic loss caused by brucellosis. Furthermore, Xie and Horan [15] investigates private responses and ecological impact of policies, which is our future research context. In summation, the brucellosis control and eradication program is multi-faceted and needs to combine several methods.

There are some limitation in our paper. Firstly, the amount of brucella discharged by the exposed and infectious should be different and an auxiliary parameter should be introduced. However, this difference between the discharge rates is hard to quantify and is also uncertain, so this paper assume that the exposed and infectious discharge the same amount of brucellosis into the environment.

In future work, we can investigate brucellosis from the following aspects: to begin with, managing the birth rate has larger influence in brucellosis spreading, but it has a close relationship with the economy. Hence, in order to discuss brucellosis comprehensively and fully, we should combine with economic factors and statistical methods. Secondly, vaccination of brucella for cows has many indeterminacies. After vaccination, resistance of herds may not be complete, and some vaccinated individual may become infected. Moreover, for dairy herds, vaccine may have some influence on milk. So, the vaccination measures need more discussion in our future work.

## Supporting Information

Appendix S1 **Table S1**, The positive data of dairy cattle from 2001 to 2010 year in some cities of Zhejiang province and the whole province. **Table S2**, The dairy cattle population from 2000 to 2009 year in Zhejiang province.
(PDF)

Figure S1
**(a)** 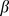 **in term of** 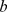 **. (b)** 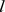 **in term of** 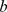 **, where** 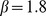 **.** The other parameter values are the same as in Fig. 5 of the main article.(EPS)

Figure S2

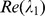
 **in term of** 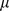 **and** 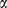 **. (a)** 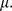 **(b)** 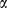 **.** 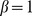 and other parameter values are the same as in Fig. 5 of the main article.(EPS)

Figure S3

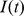
 **and** 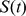 **with** 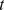 **under different** 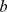 **and** 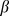 **.** (a) 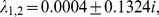 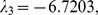 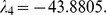 (b)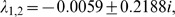 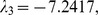 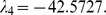 (c) 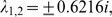 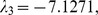 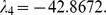 (d) 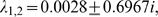 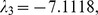 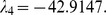 The initial values 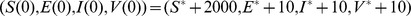.(EPS)

Figure S4

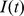
 **with** 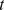 **under different parameters, where** 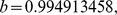 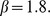 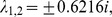 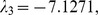 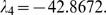

(EPS)
