# Supplementary material for: Prediction and Control of Brucellosis Transmission of Dairy Cattle in Zhejiang Province, China
Source: PLoS One. 2014 Nov 11;9(11):e108592. doi: 10.1371/journal.pone.0108592 (PMC4227660; doi:10.1371/journal.pone.0108592)
Supplement: Appendix S1 — Table S1, The positive data of dairy cattle from 2001 to 2010 year in some cities of Zhejiang province and the whole province. Table S2, The dairy cattle population from 2000 to 2009 year in Zhejiang province. (PDF) [file pone.0108592.s001.pdf]

Prediction and Control of Brucellosis Transmission of Dairy Cattle in Zhejiang Province, China

Juan Zhang[1,2,△], Gui-Quan Sun[2,3,△], Xiang-Dong Sun[4], Qiang Hou[5], Mingtao Li[2], Baoxu Huang[4], Haiyan Wang[6], Zhen Jin[2,*]
1 School of Mechatronic Engineering, North University of China, Taiyuan, Shan'xi 030051, People's Republic of China
2 Complex Systems Research Center, Shanxi University, Taiyuan, Shan'xi 030006, People's Republic of China
3 School of Mathematical Science, Fudan University, Shanghai 200433, People's Republic of China
4 The Laboratory of Animal Epidemiological Surveillance, China Animal Health & Epidemiology Center, Qingdao, Shandong 266032, People's Republic of China
5 Department of Mathematics, North University of China, Taiyuan, Shan'xi 030051, People's Republic of China
6 School of Mathematical & Natural Sciences, Arizona State University, Phoenix, AZ 85069-7100, USA
∗ **E-mail: jinzhn@263.net**
△ **These authors contributed equally to this work.**

Appendix S1

The detailed description of model (1)

In model, besides the self-sufficiency of dairy cows in Zhejiang province, we consider the import from other provinces. Naturally, at the same time there will be some infected cows to be introduced. As we mentioned in the introduction section, we consider the direction contact transmission of E and I to S, and indirection contact transmission by V to S. E and I can discharge excrement including lots of brucella to environment. The prevention and control measures we consider in model include regular detection, culling positive reactors and disinfection of environment.

The change over time in the size of the susceptible dairy cows population $S(t)$ consists of the input, which includes the external import number $(1 - c_1 - c_2)A$ and the internal birth number $bS(t)$ per unit time, and the output, which includes the natural elimination number $mS(t)$ due to low milk production and the infection number $\epsilon\beta\frac{S(t)E(t)}{N(t)} + \beta\frac{S(t)I(t)}{N(t)} + \alpha S(t)V(t)$ per unit time. Suppose A is the total number of the imported dairy cows from other provinces per unit time. Due to insufficient quarantine inspection, there must exist some infected individuals among them: the exposed and the infectious, the proportions of which are denoted by c_1, c_2. So, the number of the imported susceptible individual per unit time is $(1-c_1-c_2)A$. According to the birth rate, we suppose that once the individual is infected with brucella, it cannot give birth to a heathy baby. Even if a baby is born, it does not survive for very long. As a result, we suppose the birth number of all dairy cows per unit time is $bS(t)$. Next, we introduce the infection term, that is incidence rate, in detail. Generally, it can be written as $\beta_1\frac{C(N)I(t)S(t)}{N(t)}$. Let $C(N)$ is the number of contact with other dairy cows per susceptible individual per unit time. So, $\frac{C(N)I(t)}{N(t)}$ is the number of contact with an infectious dairy cows per susceptible individual per unit time. If the probability to be infected the epidemic after contact with a infectious individual is β_1, $\beta_1\frac{C(N)I(t)}{N(t)}$ is the infection rate for the susceptible individual, defined as the force of infection. So, $\beta_1\frac{C(N)I(t)S(t)}{N(t)}$ is the infection rate for all susceptible individuals to be infected brucellosis, that is the number of the new infected produced per unit time. Because dairy cows in Zhejiang province are on a large scale and raised in captivity form, the number of contacts with other dairy cows per individual is almost constant, that is, $C(N)$ can be seen

as a constant, denoted by C. Let $\beta = \beta_1 C$, so the transmission term is rewritten as $\beta\frac{S(t)I(t)}{N(t)}$. When a susceptible dairy cow contacts with a exposed individual, the infection rate is much lower. Therefore, we introduce a auxiliary variable ϵ $(0 < \epsilon < 1)$ and the transmission term between the susceptible and the exposed becomes $\epsilon\beta\frac{S(t)E(t)}{N(t)}$. Besides the direct contact infection between individuals, we add the indirect contact infection between the susceptible individual and the brucella in environment. For a susceptible individual, the rate to be infected is proportional to the quantity of brucella in environment. So, the indirect contact transmission term is $\alpha S(t)V(t)$. Consequently, the change over time in the size of the susceptible dairy cows population $S(t)$ is as follows.

$$\frac{dS(t)}{dt} = (1 - c_1 - c_2)A + (b - m)S(t) - \epsilon\beta\frac{S(t)E(t)}{N(t)} - \beta\frac{S(t)I(t)}{N(t)} - \alpha S(t)V(t).$$

The interpretation for the change over time in the size of the exposed dairy cows population $E(t)$ is similar. Once be infected, the individuals will enter the exposed compartment at the rate of $\epsilon\beta\frac{S(t)E(t)}{N(t)} + \beta\frac{S(t)I(t)}{N(t)} + \alpha S(t)V(t)$. The external import of dairy cows, A, enter the exposed compartment on a ratio of c_1 per unit time. Besides the natural elimination number $mE(t)$ per unit time, the exposed will show clinical symptoms at a rate of δ per unit time. Whether the exposed individual can be detectable by detection methods is still unknown, therefore we assume that it cannot be found out. Thus, the change over time in the size of the exposed dairy cows population $E(t)$ is as follows.

$$\frac{dE(t)}{dt} = c_1 A + \epsilon\beta\frac{S(t)E(t)}{N(t)} + \beta\frac{S(t)I(t)}{N(t)} + \alpha S(t)V(t) - mE(t) - \delta E(t).$$

For the infectious compartment, there are the fractional import $c_2 A$, clinical outbreak $\delta E(t)$ and natural elimination $mI(t)$. Moreover, once a infectious individual is diagnosed with brucellosis, it will be culled at rate of μ that is the product of surveillance rate and culling rate during a single test. σ is frequency of diagnosis per unit time. So, the culling number of dairy cows per unit time is $\sigma\mu I(t)$. Thus, the change over time in the size of the infectious dairy cows population $I(t)$ is as follows.

$$\frac{dI(t)}{dt} = c_2 A + \delta E(t) - mI(t) - \sigma\mu I(t).$$

For the brucella in environment, the quantity discharged by the exposed and the infected dairy cows on and on per unite time is assumed as $r(E(t) + I(t))$. w is a natural death rate of brucella per unite time. $klV(t)$ is the quantity of death of brucella due to sterilization and disinfection per unit time. k is the effective rate of sterilization for every disinfection. l is frequency of disinfection per unit time. Thus, the change over time in the size of the brucella population $V(t)$ is as follows.

$$\frac{dV(t)}{dt} = r(E(t) + I(t)) - wV(t) - klV(t).$$

To sum up, the model we will discuss is

$$\begin{cases} \frac{dS(t)}{dt} = (1 - c_1 - c_2)A + bS(t) - \epsilon\beta\frac{S(t)E(t)}{N(t)} - \beta\frac{S(t)I(t)}{N(t)} - \alpha S(t)V(t) - mS(t), \\ \frac{dE(t)}{dt} = c_1 A + \epsilon\beta\frac{S(t)E(t)}{N(t)} + \beta\frac{S(t)I(t)}{N(t)} + \alpha S(t)V(t) - mE(t) - \delta E(t), \\ \frac{dI(t)}{dt} = c_2 A + \delta E(t) - mI(t) - \sigma\mu I(t), \\ \frac{dV(t)}{dt} = r(E(t) + I(t)) - wV(t) - klV(t). \end{cases} \tag{1}$$

The calculation process of R0

For the following system

$$\begin{cases} \frac{dS(t)}{dt} = A + bS(t) - \epsilon\beta\frac{S(t)E(t)}{N(t)} - \beta\frac{S(t)I(t)}{N(t)} - \alpha S(t)V(t) - mS(t), \\ \frac{dE(t)}{dt} = \epsilon\beta\frac{S(t)E(t)}{N(t)} + \beta\frac{S(t)I(t)}{N(t)} + \alpha S(t)V(t) - mE(t) - \delta E(t), \\ \frac{dI(t)}{dt} = \delta E(t) - mI(t) - \sigma\mu I(t), \\ \frac{dV(t)}{dt} = r(E(t) + I(t)) - wV(t) - klV(t), \end{cases}$$

only when $m > b$, the disease-free equilibrium exists and it can be given as follows

$$P_0 = (S^0, 0, 0, 0),$$

where $S^0 = \frac{A}{m-b}$.

Having the disease-free equilibrium and applying the next-generation method (see [?, ?]), firstly we give

$$\mathscr{F} = \begin{pmatrix} \frac{\epsilon\beta S(t)E(t)}{N(t)} + \frac{\beta S(t)I(t)}{N(t)} + \alpha S(t)V(t) \\ 0 \\ 0 \end{pmatrix}$$

and

$$\mathscr{V} = \begin{pmatrix} mE(t) + \delta E(t) \\ mI(t) + \sigma\mu I(t) - \delta E(t) \\ wV(t) + klV(t) - r(E(t) + I(t)) \end{pmatrix}.$$

Then,

$$F = \begin{pmatrix} \epsilon\beta + \beta + \alpha S(t) & 0 & 0 \\ 0 & 0 & 0 \\ 0 & 0 & 0 \end{pmatrix}$$

and

$$V = \begin{pmatrix} m+\delta & 0 & 0 \\ -\delta & m+\sigma\mu & 0 \\ -r & -r & w+kl \end{pmatrix}.$$

According to $R_0 = \rho(FV^{-1})$, the basic reproduction number of the system is

$$R_0 = \frac{\epsilon\beta}{m+\delta} + \frac{\beta\delta}{(m+\delta)(m+\sigma\mu)} + \frac{\alpha S^0 r(m+\delta+\sigma\mu)}{(m+\delta)(m+\sigma\mu)(w+kl)}.$$

Obviously, from the expression of the basic reproduction number, we can see that it includes three parts. The first part is the number of secondary cases infected by the exposed, the second part by the infectious, the last part by the brucella in environment during the entire infectious period.

The dynamical behavior of model (1)

Theorem 1 *For system (1), there is only one endemic equilibrium P^*.*

Proof. Let $P^* = (S^*, E^*, I^*, V^*)$ satisfy the following equations:

$$\begin{cases} (1 - c_1 - c_2)A + bS^* - \epsilon\beta\frac{S^*E^*}{N^*} - \beta\frac{S^*I^*}{N^*} - \alpha S^*V^* - mS^* = 0, \\ c_1A + \epsilon\beta\frac{S^*E^*}{N^*} + \beta\frac{S^*I^*}{N^*} + \alpha S^*V^* - mE^* - \delta E^* = 0, \\ c_2A + \delta E^* - mI^* - \sigma\mu I^* = 0, \\ r(E^* + I^*) - wV^* - klV^* = 0. \end{cases} \tag{2}$$

Obviously,

$$\begin{aligned} E^* &= \frac{(m+\sigma\mu)I^* - c_2A}{\delta}, N^* = \frac{bS^* + A - \sigma\mu I^*}{m}, \\ V^* &= \frac{r[(m+\sigma\mu+\delta)I^* - c_2A]}{\delta(w+kl)}. \end{aligned} \tag{3}$$

Applying $S^* + E^* + I^* = N^*$, we have

$$\begin{aligned} S^* + \frac{(m+\sigma\mu)I^* - c_2A}{\delta} + I^* &= \frac{bS^* + A - \sigma\mu I^*}{m} \\ (m-b)\delta S^* = (mc_2+\delta)A - (m+\delta)(m+\sigma\mu)I^* \end{aligned} \tag{4}$$

Case 1. When $b = m$, $I^* = \frac{A(\delta+c_2m)}{(m+\sigma\mu)(m+\delta)}$, $E^* = \frac{A(1-c_2)}{m+\delta}$, $V^* = \frac{Ar[m+\delta+\sigma\mu(1-c_2)]}{(w+kl)(m+\sigma\mu)(m+\delta)}$, substituting E^*, I^*, V^* into the second equation of (2), we have

$$a_1S^{*2} + a_2S^* + a_3 = 0, \tag{5}$$

where

$$\begin{aligned} a_1 &= \alpha V^* > 0, \\ a_2 &= c_1A + \epsilon E^* + \beta I^* + \alpha V^*(E^* + I^*) - (m+\delta)E^*, \\ a_3 &= [c_1A - (m+\delta)E^*](E^* + I^*) = (c_1 + c_2 - 1)A(E^* + I^*) < 0. \end{aligned}$$

Consequently, equation (5) has only one positive solution S^*, that is, there is only one positive equilibrium $P_* = (S^*, E^*, I^*, V^*)$.

Case 2. When $b \neq m$, from (4), we have

$$S^* = \frac{(mc_2+\delta)A - (m+\delta)(m+\sigma\mu)I^*}{(m-b)\delta}. \tag{6}$$

Substituting (3) and (6) into the second equation in system(2), we have

$$\begin{aligned} f(I^*) = \quad & c_1A + \frac{\epsilon\beta m h_1(I^*)h_2(I^*)}{\delta\{bh_1(I^*) + h_3(I^*)\}} + \frac{\beta m h_1(I^*)I^*}{bh_1(I^*) + h_3(I^*)} + \frac{\alpha h_1(I^*)r[h_2(I^*) + \delta I^*]}{\delta(w+kl)} \\ & - \frac{(m+\delta)h_2(I^*)}{\delta} = 0 \end{aligned} \tag{7}$$

where $h_1(I^*) = (mc_2+\delta)A - (m+\delta)(m+\sigma\mu)I^*$, $h_2(I^*) = (m+\sigma\mu)I^* - c_2A$ and $h_3(I^*) = (A - \sigma\mu I^*)(m-b)\delta$.

If $b < m$, to ensure that $P^* = (S^*, E^*, I^*, V^*)$ is positive equilibrium, it must hold that

$$\begin{aligned} S^* > 0 \quad &\Leftrightarrow \quad I^* < \frac{A(mc_2+\delta)}{(m+\delta)(m+\sigma\mu)}, \\ E^* > 0 \quad &\Leftrightarrow \quad I^* > \frac{Ac_2}{m+\sigma\mu}, \\ V^* > 0 \quad &\Leftrightarrow \quad I^* > \frac{Ac_2}{m+\sigma\mu+\delta}, \\ N^* > 0 \quad &\Leftrightarrow \quad I^* < \frac{Am(bc_2+\delta)}{b(m+\delta)(m+\sigma\mu) + (m-b)\delta\sigma\mu}. \end{aligned}$$

So, we should make sure that $\frac{Ac_2}{m+\sigma\mu} < I^* < \frac{A(mc_2+\delta)}{(m+\delta)(m+\sigma\mu)}$. Let $I_1 = \frac{Ac_2}{m+\sigma\mu}$ and $I_2 = \frac{A(mc_2+\delta)}{(m+\delta)(m+\sigma\mu)}$.

It is easy to get that

$$\begin{aligned} f(I_1) &= c_1A + \frac{m\beta(1-c_2)c_2A}{(m+\sigma\mu)b(1-c_2)+(m-b)[m+\sigma\mu(1-c_2)]} + \frac{\alpha\delta A^2(1-c_2)rc_2}{(m-b)\delta(m+\sigma\mu)(w+kl)} > 0, \\ f(I_2) &= A(c_1+c_2-1) < 0. \end{aligned}$$

Because the coefficient of the highest-order (third-order) term of $f(I^*)$, that is $(m+\sigma\mu)(m+\delta)\alpha rm(mb+\delta b+\sigma\mu b+\sigma\mu\delta)(m+\sigma\mu+\delta)$, is positive, $f(I_1) > 0$ and $f(I_2) < 0$, there must exist a unique positive $I_1 < I^* < I_2$ such that $f(I_*) = 0$.
If $b > m$, to ensure that $P^* = (S^*, E^*, I^*, V^*)$ is positive equilibrium, it must hold that

$$\begin{aligned} S^* > 0 &\Leftrightarrow I^* > \frac{A(mc_2+\delta)}{(m+\delta)(m+\sigma\mu)}, \\ E^* > 0 &\Leftrightarrow I^* > \frac{Ac_2}{m+\sigma\mu}, \\ V^* > 0 &\Leftrightarrow I^* > \frac{Ac_2}{m+\sigma\mu+\delta}, \\ N^* > 0 &\Leftrightarrow I^* > \frac{Am(bc_2+\delta)}{b(m+\delta)(m+\sigma\mu)+(m-b)\delta\sigma\mu}. \end{aligned}$$

So, we have $I^* > I_2 > I_1$. Because $f(I_2) < 0$, $f(I_1) > 0$, and the coefficient of the highest-order term of $f(I^*)$, that is $(m+\sigma\mu)(m+\delta)\alpha rm(mb+\delta b+\sigma\mu b+\sigma\mu\delta)(m+\sigma\mu+\delta)$, is positive, there must exist a unique positive solution $I^* > I_2$ such that $f(I^*) = 0$.
■

Considering the system (1), it is known that if there is the infected dairy cows to import, the disease cannot disappear. So, what will the situation of the brucellosis be if there exists no import of the infectious cows?

Let $c_1 = c_2 = 0$, we have

$$\begin{cases} \frac{dS(t)}{dt} = A + bS(t) - \epsilon\beta\frac{S(t)E(t)}{N(t)} - \beta\frac{S(t)I(t)}{N(t)} - \alpha S(t)V(t) - mS(t), \\ \frac{dE(t)}{dt} = \epsilon\beta\frac{S(t)E(t)}{N(t)} + \beta\frac{S(t)I(t)}{N(t)} + \alpha S(t)V(t) - mE(t) - \delta E(t), \\ \frac{dI(t)}{dt} = \delta E(t) - mI(t) - \sigma\mu I(t), \\ \frac{dV(t)}{dt} = r(E(t)+I(t)) - wV(t) - klV(t). \end{cases} \tag{8}$$

Theorem 2 *For the system (8), we have the following results:*
(1) When $m > b$ and $R_0 < 1$, the system (8) only has the disease-free equilibrium P^0.
(2) When $m > b$ and $R_0 \geq 1$, the system (8) has the disease-free equilibrium P^0 and only one positive equilibria P^*.
(3) When $m \leq b$, the system (8) has the only one positive equilibrium P^*.

Proof. Now, we discuss the number of the positive equilibria under different conditions.
Let

$$\begin{cases} A + bS - \epsilon\beta\frac{SE}{N} - \beta\frac{SI}{N} - \alpha SV - mS = 0, \\ \epsilon\beta\frac{SE}{N} + \beta\frac{SI}{N} + \alpha SV - mE - \delta E = 0, \\ \delta E - mI - \sigma\mu I = 0, \\ r(E+I) - wV - klV = 0. \end{cases} \tag{9}$$

Easily,

$$
\begin{aligned}
E^* &= \frac{(m+\sigma\mu)}{\delta} I^*, \\
V^* &= \frac{r(m+\sigma\mu+\delta)}{\delta(w+kl)} I^*, \\
N^* &= \frac{A+bS^*-\sigma\mu I^*}{m}.
\end{aligned} \tag{10}
$$

According to (10) and $S^*+E^*+I^*=N^*$, it can be obtained that

$$
S^* + \frac{(m+\sigma\mu)}{\delta} I^* + I^* = \frac{A+bS^*-\sigma\mu I^*}{m}. \tag{11}
$$

Case 1. $m=b$.
When $m=b$, we can calculate that

$$
\begin{aligned}
I^* &= \frac{A\delta}{(m+\sigma\mu)(m+\delta)}, \\
E^* &= \frac{A}{m+\delta}, \\
V^* &= \frac{Ar(m+\sigma\mu+\delta)}{(w+kl)(m+\sigma\mu)(m+\delta)}.
\end{aligned}
$$

From the first equation in system (9), we have

$$
\alpha b V^* S^{*2} + [\beta m(\epsilon E^* + I^*) + \alpha V^*(A-\sigma\mu I^*) - Ab] S^* + A(\sigma\mu I^* - A) = 0.
$$

Because $\alpha b V^* > 0$, $A(\sigma\mu I^* - A) < 0$, the above equation has only positive solution. So, when $m=b$, It is easy to know that the system has no disease-free equilibrium and there is only one positive equilibrium $P^* = (S^*, E^*, I^*, V^*)$.

Case 2. $m \neq b$.

When $m \neq b$, it can be obtained by equations (11):

$$
S^* = \frac{A\delta - (m+\sigma\mu)(m+\delta)I^*}{(m-b)\delta}. \tag{12}
$$

Substituting (10) and (12) into the second equation in system (9), we have

$$
(x_1 I^{*2} + x_2 I^* + x_3) I^* = 0, \tag{13}
$$

where

$$
\begin{aligned}
a &= (m+\sigma\mu)(m+\delta), \\
c &= w+kl, \\
x_1 &= \alpha(m+\sigma\mu+\delta)a[ab+\sigma\mu\delta(m-b)] > 0, \\
x_2 &= -[\alpha r\delta A(m+\sigma\mu+\delta)(ab+\sigma\mu\delta(m-b)) + (m-b)^2 ac\delta(a-\sigma\mu\delta) \\
&\quad + a^2 m\delta(m-b)c(R_0-1)], \\
x_3 &= A\delta^2 mac(R_0-1)(m-b).
\end{aligned} \tag{14}
$$

The case when $I^*=0$ has been discussed. Now, we consider $x_1 I^{*2} + x_2 I^* + x_3 = 0$.

(i) $m > b$.

When $m > b$ and $R_0 < 1$, then $x_3 < 0$ and the system have two solutions: one is positive and the other is negative. If $R_0 = 1$, then $x_3 = 0$ and $x_2 = -[\alpha r\delta A(m+\sigma\mu+\delta)(ab+\sigma\mu\delta(m-b))+(m-b)^2 ac\delta(a-\sigma\mu\delta)] < 0$, that is the system has a zero solution and a positive solution. If $R_0 > 1$, then $x_3 > 0$. In that case, we need to judge the sign symbol of $\triangle = x_2^2 - 4x_1x_3$. If $\Delta > 0$, which is proved as follows, there are two positive solution.

(ii) $m < b$.

When $m < b$ and $R_0 > 1$, then $x_3 < 0$ and the system have two solutions: one is positive and the other is negative. If $R_0 = 1$, then $x_3 = 0$ and $x_2 = -[\alpha r\delta A(m+\sigma\mu+\delta)(ab+\sigma\mu\delta(m-b))+(m-b)^2 ac\delta(a-\sigma\mu\delta)] < 0$, that is the system has a zero solution and a positive solution. If $R_0 < 1$, then $x_3 > 0$. Now we need to judge the sign of $\Delta = x_2^2 - 4x_1x_3$.

Now, we prove $\Delta = x_2^2 - 4x_1x_3 > 0$. Substituting (14) into Δ, it can be obtained

$$\Delta = y_1(R_0 - 1)^2 + y_2(R_0 - 1) + y_3, \tag{15}$$

where

$$\begin{aligned} y_1 &= a^4c^2\delta^2m^2(b-m)^2, \\ y_2 &= 2a^2c\delta^2m(m-b)[ac(a-\delta\mu\sigma)(b-m)^2 - A\alpha r(ab-\delta\mu\sigma(b-m))(m+\delta+\sigma\mu)], \\ y_3 &= [ac\delta(a-\delta\mu\sigma)(b-m)^2 + A\alpha\delta r(ab-\delta\mu\sigma(b-m))(m+\delta+\sigma\mu)]^2. \end{aligned}$$

Because $y_1 > 0$, $y_3 > 0$ and $y_2^2 - 4y_1y_3 = 16Aa^5\alpha c\delta^4m^2r(b-m)^4(a-\delta\mu\sigma)(m+\delta+\sigma\mu)(\delta\mu\sigma(b-m)-ab) < 0$, so Δ is always positive.

By observing (12), $I^* > 0$ cannot promise $S^* > 0$. So, we also need to discuss the sign symbol of S^*. Substituting (12) into (13), it can be obtained

$$z_1 S^{*2} + z_2 S^* + z_3 = 0, \tag{16}$$

where

$$\begin{aligned} z_1 &= x_1(m-b)^2\delta^2 > 0, \\ z_2 &= -(2A\delta x_1 + ax_2\delta)(m-b)\delta, \\ z_3 &= x_1A^2\delta^2 + ax_2A\delta + a^2x_3 = -A\delta^2a^2c(m-b)^2(a-\mu\sigma\delta) < 0. \end{aligned}$$

So, equation (16) has only one positive solution.

Combining the situations of S^* and I^*, we can find that when $m > b$, $R_0 < 1$ or $m < b$, $R_0 > 1$, there are two positive I^*, but there is only one positive S^*, that is, for one solution of I^*, corresponding value of S^* should be negative. So, there is single positive equilibrium P^*. The proof is completed.
■

Theorem 3 *When $m > b$ and $R_0 < 1$, then the disease-free equilibrium P^0 of system (8) is globally asymptotically stable.*

Proof. By [?, ?, ?], we know that P^0 is local asymptotically stable. Now we define a Lyapunov function

$$L = \frac{w+kl}{\alpha S^0}E + \frac{(w+kl)\beta + \alpha S^0 r}{\alpha S^0(m+\sigma\mu)}I + V \geq 0.$$

When $m > b$ and $R_0 < 1$, the Lyapunov function satisfies

$$\begin{aligned} \dot{L} &= \frac{w+kl}{\alpha S^0}\dot{E} + \frac{(w+kl)\beta + \alpha S^0 r}{\alpha S^0(m+\sigma\mu)}\dot{I} + \dot{V} \\ &= \frac{(m+\delta)(w+kl)}{\alpha S^0}(R_0 - 1)E \\ &\leq 0. \end{aligned}$$

Moreover, $\dot{L} = 0$ only hold when $E = 0$. It is easy to conclude that the disease-free equilibrium point P^0 is the only fixed point of the system. Hence applying the Lyapunov-LaSalle asymptotic stability theorem in [?, ?], the disease-free equilibrium point P^0 is globally asymptotically stable.
■

Theorem 4 *For the system (8), when $m > b$ and $R_0 \geq 1$ or $b \geq m$, the system is uniformly persistent.*

Proof. When $m > b$, $\frac{dN}{dt} = A + bS - mN - \sigma\mu I < A + bN - mN$. We can give the positive invariant set $\Gamma = \{(S, E, I, V) \in \mathbb{R}_+^4 : 0 \leq S + E + I \leq \frac{A}{m-b}\}$. Then, $\partial\Gamma$ and Γ^0 are denoted as the boundary of Γ and the interior of Γ respectively. Firstly, let $\Phi(x_0)$ be the solution of system with the initial $x_0 = (S(0), E(0), I(0), V(0))$. Let

$$F(t) = \begin{pmatrix} \epsilon\beta & \beta & \alpha S^0 \\ 0 & 0 & 0 \\ 0 & 0 & 0 \end{pmatrix}$$

and

$$V(t) = \begin{pmatrix} m + \delta & 0 & 0 \\ -\sigma & m + \sigma\mu & 0 \\ -r & -r & w + kl \end{pmatrix}.$$

When $m > b$ and $R_0 \geq 1$, it can be easy to know that $\mathcal{S}(F - V) > 0$ by Theorem 2 in [?]. We can choose $\xi > 0$ small enough such that $\mathcal{S}(F - V - M_\xi) > 0$, where

$$M_\epsilon = \begin{pmatrix} 0 & 0 & 2\frac{\epsilon\beta\xi}{S^0+\xi} \\ 0 & 0 & 2\frac{\beta\xi}{S^0+\xi} \\ 0 & 0 & 0 \end{pmatrix}.$$

Now we prove that

$$\limsup_{m\to\infty} d(\Phi(x_0, P^0) \geq \delta, x_0 \in \Gamma^0.$$

Suppose, by contradiction, there exists $T > 0$ such that

$$\limsup_{m\to\infty} d(\Phi(x_0, P^0) < \delta, x_0 \in \Gamma^0$$

for $t > T$. To take $\Phi(x_0, P^0)$ as initial condition, we can have

$$\limsup_{m\to\infty} d(\Phi(x_0, P^0) < \delta, x_0 \in \Gamma^0$$

for all $t > 0$, which implies that $S^0 - \xi \leq S(t) \leq S^0 + \xi$. Then

$$\begin{aligned} \frac{dE}{dt} &\geq \epsilon\beta(1 - \frac{2\xi}{S^0+\xi})E + \beta(1 - \frac{2\xi}{S^0+\xi})I + \alpha(S^0 - \xi)V - mE - \delta E, \\ \frac{dI}{dt} &\geq \delta E - mI - \sigma\mu I, \\ \frac{dV}{dt} &\geq r(E + I) - wV - klV. \end{aligned} \tag{17}$$

Note that $\mathcal{S}(F - V - M_\xi) > 0$. It then follows that any solution of (17) with positive initial value $x_0 \in \Gamma^0$ goes to infinity as $t \to \infty$. By the comparison theorem [?], we know that $E(t) \to \infty, I(t) \to \infty, V(t) \to \infty$ for $t \to \infty$. Thus,

$$\limsup_{m\to\infty} d(\Phi(x_0, P^0) \geq \delta, x_0 \in \Gamma^0$$

holds. So, the system is weekly uniformly persistent. It is easy to know that Γ and Γ^0 are positive invariant sets and the system is point dissipative. Thus, by the Theorem 4.2 in [?], when $m > b$ and $R_0 \geq 1$, we can obtain that the system is uniformly persistent.

When $m < b$, there is only a positive equilibrium. So the system must be uniformly persistent and the disease cannot disappear. ■

In the real case, due to constraint of sensibility of detection methods, as long as there are dairy cows to import, the input of infected dairy cows is unavoidable. In this case, brucellosis cannot disappear in Zhejiang province. Then if there are no dairy cows imported and self-support is only permitted to exist, what will the situation of brucellosis be? Let $A = 0$, we have the following system:

$$\begin{cases} \frac{dS(t)}{dt} = bS(t) - \epsilon\beta\frac{S(t)E(t)}{N(t)} - \beta\frac{S(t)I(t)}{N(t)} - \alpha S(t)V(t) - mS(t), \\ \frac{dE(t)}{dt} = \epsilon\beta\frac{S(t)E(t)}{N(t)} + \beta\frac{S(t)I(t)}{N(t)} + \alpha S(t)V(t) - mE(t) - \delta E(t), \\ \frac{dI(t)}{dt} = \delta E(t) - mI(t) - \sigma\mu I(t), \\ \frac{dV(t)}{dt} = r(E(t) + I(t)) - wV(t) - klV(t). \end{cases} \tag{18}$$

It is easy to know that in the absence of disease, the total dairy cows population size $N(t)$ will decline to 0 if $b < m$. If $b = m$, the population size will remain constant, and if $b > m$, the population size will grows exponentially. However, if there exists the disease, the situations will be complicated and the results are shown as following Theorems.

Theorem 5 *For the system (18), we have the following results:*
(1) if $b < m$, there is a zero equilibrium $O = (0, 0, 0, 0)$.
(2) if $b = m$, there are a zero equilibrium $O = (0, 0, 0, 0)$ and a disease-free equilibrium $P^0 = (S^0, 0, 0, 0)$, where S^0 is a number that depends on the initial value.
(3) if $b > m$ and $(mb+\delta b+\sigma\mu b+\sigma\mu\delta)-\epsilon\beta(m+\sigma\mu)-\beta\delta > 0$, there is a zero equilibrium $O = (0, 0, 0, 0)$ and only one endemic equilibrium $P^* = (S^*, E^*, I^*, V^*)$, where

$$\begin{aligned} S^* &= \frac{(m+\sigma\mu)(m+\delta)I^*}{(b-m)\delta}, \\ E^* &= \frac{(m+\sigma\mu)I^*}{\delta}, \\ V^* &= \frac{r(m+\sigma\mu+\delta)I^*}{\delta(w+kl)}, \\ I^* &= \frac{(b-m)\delta(w+kl)[(mb+\delta b+\sigma\mu b+\sigma\mu\delta)-\epsilon\beta(m+\sigma\mu)-\beta\delta]}{\alpha r(m+\sigma\mu+\delta)(mb+\delta b+\sigma\mu b+\sigma\mu\delta)}. \end{aligned}$$

Theorem 6 *For the system (18), we have the following results:*
(1) if $b < m$, the zero equilibrium $O = (0, 0, 0, 0)$ is stable.
(2) if $b = m$, the disease-free equilibrium $P^0 = (S^0, 0, 0, 0)$, where S^0 is a number that depends on the initial value, is stable.

Proof. (1) When $b < m$, the general solution of the first equation of the system can be given as follows.

$$S(t) = S(0)\exp\big(-\int_0^t \epsilon\beta\frac{E(t)}{N(t)} + \beta\frac{I(t)}{N(t)} + \alpha V(t) + (m-b)dt\big).$$

It is easy to know that when $b < m$, $\int_0^t (m-b)dt \to +\infty$ as $t \to +\infty$. So $S(t) \to 0$ as $t \to +\infty$. So, the zero equilibrium $O = (0, 0, 0, 0)$ is stable.

(2) When $b=m$, the first equation of the system can be written as follows.

$$\frac{dS(t)}{dt}=-(\epsilon\beta\frac{E(t)S(t)}{N(t)}+\beta\frac{I(t)S(t)}{N(t)}+\alpha V(t)S(t))\leq 0.$$

Because $S(t)$ is monotone decreasing with zero as lower bound. So $E(t), I(t), V(t)\to 0$, $S(t)\to S^0 = S(0)\exp\big(-\int_0^\infty \epsilon\beta\frac{E(t)}{N(t)}+\beta\frac{I(t)}{N(t)}+\alpha V(t)dt\big)$ as $t\to+\infty$. ■

Proposition 1 *According to the equilibrium E_*, it can be stable conditionally, otherwise it is not stable. Under some conditions, there will appear Hopf Bifurcation, that is, the system (18) can have periodic solution.*

Firstly, let us look at the influence of parameters on $Re(\lambda_1)$, the real part of the eigenvalues of Jacobian matrix at the equilibrium E_*. The sign symbol of $Re(\lambda_1)$ determines the local behavior of $P^*=(S^*,E^*,I^*,V^*)$. If $Re(\lambda_1)<0$, E_* is local stable. If $Re(\lambda_1)=0$, there will appear periodic cycle. If $Re(\lambda_1)>0$, E_* is unstable. By the numerical calculating, it can be found that b and β have the biggest effects on $Re(\lambda_1)$, followed by l. μ is the least and α has nothing to do with $Re(\lambda_1)$. We can see the regions of $Re(\lambda_1)>0$ and $Re(\lambda_1)<0$ in term of b, β and l, respectively, in Fig.S1, where the curves represent $Re(\lambda_1)=0$. From Fig.S1(a), we can see that when $\beta<0.7$, $Re(\lambda_1)$ always is positive. From Fig.S1(b), we can see that when $\beta=1.8$ and $b<0.7$, $Re(\lambda_1)$ always is negative for all positive l, that is, P^* is local asymptotically stable.

Because the influence of μ is smaller, we only draw three curves of $Re(\lambda_1)$ in term of μ under different b. It can be observed from Fig.S2(a) that when $b>0.8$, the change of μ cannot change the sign symbol of $Re(\lambda_1)$ for its effect is very small. In Fig.S1(a), the point $Re(\lambda_1)$ with $b=0.5$ and $\beta=1$ is near the curve, so in Fig.S2(a), changing μ can make the curve to cross $Re(\lambda_1)=0$. From Fig.S2(b), we can see that α has no influence on $Re(\lambda_1)$.

Figs.S3 show phase diagrams of $I(t)$ and $S(t)$ with time under different b and β, respectively, which are used to confirm above results. From the Fig.S3(b), when $b=0.5$ and $b=1.8$, $Re(\lambda_1)=-0.0059<0$ and the solution curve tends to the P^*. So, the equilibrium P^* is stable. From the Figs.S3(a) and (d), $Re(\lambda_1)>0$, which shows that the system lose stability, and the radius of the solution curve becomes bigger and bigger and is far from the P^*. Moreover, in the Fig.S3(c), $Re(\lambda_1)=0$ and there appear periodic cycles.

Applying the eigenvalues of Jacobian matrix, we only obtain the behavior of solutions when the initial values is near E_*, that is the local character of E_*. In order to observe clearly the global characters, we show the $I(t)$ with t under very different initial values, where parameter values is the same as the Fig.S3(c). From Fig.S4, we can see that under different initial values, the tendency of the solution curves are different. When $(S(0),E(0),I(0),V(0))=(S^*+4000,E^*+10,I^*+20,V^*+10)$, the solution is a periodic cycle. However, when the initial values $(S(0),E(0),I(0),V(0))=(10000,100,400,100)$, the solution has quasi-periodic characteristics. There would appear chaos when the initial values $(S(0),E(0),I(0),V(0))=(5000,10,40,10)$. It can be concluded thus that with different initial values, the tendency and character of the solution curves are very different, that is, the global character of E_* is complicated.

The data of dairy cattle in Zhejiang province

The positive data are listed in Table S1 and dairy cattle population are listed in Table S2.

Table S 1. The positive data of dairy cattle from 2001 to 2010 year in some cities of Zhejiang province and the whole province.

province/city	2001	2002	2003	2004	2005	2006	2007	2008	2009	2010
Zhejiang province	14	31	30	37	248	225	69	173	527	454
Hangzhou city	0	3	2	37	171	125	6	0	31	0
Ningbo city	14	28	0	0	24	8	21	16	15	0
Huzhou city	0	0	0	0	24	0	0	0	52	0
Zhoushan city	0	0	2	0	3	13	0	0	0	0

Table S 2. The dairy cattle population from 2000 to 2009 year in Zhejiang province.

province	2000	2001	2002	2003	2004	2005	2006	2007	2008	2009
Zhejiang (thousands)	40	56	67	77	79.6	80.4	76.1	52.6	65	61

REFERENCES

References

1. Diekmann O, Heesterbeek JAP, Roberts MG (2010) The construction of next-generation matrices for compartmental epidemic models. *J Royal Soc Interface* 7: 873-885.

2. van den Driessche P, Watmough J (2002) Reproduction numbers and sub-threshold endemic equilibria for compartmental models of disease transmission. *Math Biosci* 180: 29-48.

3. Diekmann O, Heesterbeek JAP, Roberts MG (1990) On the definition and the computation of the basic reproduction ratio R_0 in models for infectious diseases in heterogeneous populations. *J Math Biol* 28: 365-382.

4. LaSalle J, Lefschetz S (1961) Stability by Liapunov's direct method.*Academic, New York.*

5. Barbashin EA (1970) Introduction to the theory of stability.*Wolters-Noordhoff, Groningen.*

6. Smith H, Waltman P (1995) The theory of the chemostat. *Cambridge: Cambridge University Press.*

7. Freedman HL, Ruan SG, Tang Mx (1994) Uniform persistence and flows near a closed positively invariant set. *Journal of Dynamics and Differential Equations* 6(4): 583-600.
